# Parenting Practices and Emotional Regulation in Children with Autism Spectrum Disorder: A Mediated Moderation Model of Sibling Prosocial Behavior and Gender

**DOI:** 10.3390/ejihpe16020020

**Published:** 2026-02-03

**Authors:** Muhammad Imran, Umaira Iftikhar, Arooj Arshad, Komal Hassan, Norah Almusharraf

**Affiliations:** 1 Educational Research Lab, Prince Sultan University, Rafa Street, Riyadh 11586, Saudi Arabia; nmusharraf@psu.edu.sa; 2 Department of English, Khazar University, Baku AZ1096, Azerbaijan; 3 Riphah Institute of Clinical and Professional Psychology, Riphah International University, Lahore Gulberg Campus, Islamabad 46000, Pakistan; umairaiftikhar156@gmail.com (U.I.); arooj.arshad@riphah.edu.pk (A.A.); komal.hassan@riphah.edu.pk (K.H.)

**Keywords:** autism spectrum disorder, parenting styles, pro-social behavior, caregivers, emotional regulation

## Abstract

Children with autism spectrum disorder (ASD) frequently struggle with emotion regulation, which can be influenced by parental practices and the supportive role of siblings in encouraging emotional and social development. The study aimed to examine the relationship between parenting practices and emotional regulation of children with ASD and to explore the mediating role of the prosocial behavior of siblings between parenting practices and emotional regulation in children with ASD. Additionally, this study investigated the moderating role of sibling gender in the relationship between prosocial behavior and emotional regulation. A total of 600 parents/caregivers aged 25–40 years (*M* = 32.91, *SD* = 4.23) of children with ASD were selected from special education institutes in Lahore, Pakistan, using a non-probability, purposive sampling method. Although the majority of respondents were mothers (94.5%), the term parenting practices is used to reflect a family-level caregiving construct rather than exclusively maternal behavior. Data were interpreted through IBM SPSS Statistics 23 and PROCESS macros, revealing that authoritative parenting had a significant positive relation with emotional regulation in children with ASD. Results also indicated that the prosocial behavior of siblings partially mediated the relationship between authoritative parenting and emotional regulation in children with ASD. Furthermore, sibling gender significantly moderated the indirect effect, with female siblings showing stronger facilitation of emotional regulation through prosocial behaviors compared to male siblings.

## 1. Introduction

Autism spectrum disorder (ASD) is a neurodevelopmental disorder characterized by repetitive behaviors and persistent difficulties with social interactions and social communication. This disorder is defined by repetitive, stereotyped, and restricted patterns of behavior, activities, and interests, as well as difficulties with social interactions and communication (Hodges et al., 2020). ASD cannot be diagnosed by a single behavior, and most children do not show all the signs (Lord et al., 2018; Aygun et al., 2024).

Emotion regulation (ER) is the automatic or intentional modulation of emotional states to support adaptive or goal-directed behavior (Cibralic et al., 2019). Several ASD characteristics impede ER effectiveness. First, alexithymia, the inability to identify, distinguish and express emotions, occurs frequently among individuals diagnosed with Autism Spectrum Disorder (ASD) (Bloch et al., 2021; Davidson & Morales, 2022). The expression of emotions is key to successful emotion regulation (ER), especially in situations involving cooperation and the sharing of emotions among individuals. Furthermore, communication and language deficits present in ASD may affect regulatory ability development since language competence is positively correlated with emotional competence in typical development (Cibralic et al., 2024). It is also important to consider ER deficits when addressing severe behavioral disorders and mental health comorbidities associated with ASD, as it is possible that most internalizing and externalizing symptoms in ASD stem from dysregulation of emotions and that dysregulation of emotions will worsen problems with attention, communication, problem-solving, and social interaction (Cibralic et al., 2019).

Children with ASD grow up in family systems (Bugental & Johnston, 2000; Bronfenbrenner, 1979), and the parents are responsible for managing the behaviors and deficits of their children while also acting as the primary treatment contact (Coolican et al., 2010; Minjarez et al., 2011). Families raising a child with ASD face numerous challenges and strains that are unique to families with children with ASD; rigid schedules of activities due to transition difficulties, social isolation stemming from the behavioral concerns of the child with ASD, and the conflicting demands of caregiving responsibilities with work obligations and financial responsibilities (Hutton & Caron, 2005; Montes & Halterman, 2007; Myers et al., 2009). These challenges create significant psychological stress for parents, which affects the mental health, social well-being, and overall functioning of the family (Gray, 2006).

Parenting practices are significantly related to child development, and parent–child interactions can be characterized using three distinct styles of parenting. Authoritative parenting combines high warmth and responsiveness to the needs of the child with clear boundaries and consistent expectations. For example, authoritative parents explain behavioral rules to their children while encouraging open communication and independence. Authoritarian parenting places emphasis on strict control and obedience with minimal warmth and flexibility; these parents enforce rules rigidly with limited explanation or negotiation. However, permissive parenting provides high warmth, but minimal structure or discipline; they are lenient with few consistent demands or behavioral limits. Research has shown that mindful parenting (the understanding of children’s needs, encouragement of independence and acceptance of maladaptive behaviors with compassion) is similar to authoritative parenting (Duncan et al., 2009). Research indicates that school-aged children with ASD in Taiwan were subjected to less affection, more over-protection, and greater authoritarian control from their parents (Ren et al., 2020), emphasizing the significance of parenting style in the ASD context.

Parental behavior influences prosocial behavior development in children through imitation of parental actions such as sharing, helping, and displaying empathy (El-Dakhs et al., 2024; Spinrad et al., 2019; Rashid et al., 2020). Grown-up experiences with an ASD sibling can provide increased compassion and sensitivity in typically developing (TD) siblings, who may be more patient and tolerant (Chu et al., 2023). However, compared to typical sibling relationships, the interactions between children with ASD and TD siblings show less prosocial behavior (Orm et al., 2022). Research has demonstrated that children with ASD who have siblings display better social functioning than only children with ASD, possibly due to older siblings serving as role models or possibly due to reduced parental stress (Alotaibi et al., 2025; Ben-Itzchak et al., 2019). While sibling presence benefits children with ASD, specific characteristics, particularly sibling gender, may further influence support effectiveness in promoting emotional regulation.

According to a retrospective study, children with ASD siblings performed better on tests of social functioning than ASD children who grew up as only children in their families. The advantage of older siblings for children with ASD in terms of social functioning can be attributed to two things: Either the older siblings serve as role models for their younger sibling with ASD, taking the lead in the relationship and facilitating participation in social interactions, or the older siblings have minimal stress on them as parents (Ben-Itzchak et al., 2019; Alotaibi et al., 2025). While the presence of siblings has been established as beneficial for children with ASD, the specific characteristics of these siblings, particularly their gender, may further influence the nature and effectiveness of their support in promoting emotional regulation.

Research concerning family dynamics and development in children with Autism Spectrum Disorder (ASD), has recently identified a need to consider the gender of siblings as a factor when evaluating the degree of prosocial behavior exhibited by these siblings and their potential for providing caregiving and emotional support to their siblings with autism. Research has shown that there is a difference in how men and women help others; as well as in how they provide care to other family members. This gender difference in providing care to one another may allow children with autism to have an easier time controlling their emotions when it comes to their brothers/sisters who also have autism. Cuskelly and Gunn (2006) have demonstrated through research that female siblings display much more prosocial behavior and demonstrate more empathy toward their siblings with autism than their male counterparts. It was stated in these studies that it is likely that the gender difference would result in a female sibling being able to give her brother/sister with autism better emotional support.

Orsmond and Seltzer (2007) conducted research on the relationship between a child with a developmental disability and his/her sibling who does not have a developmental disability. They found that the sisters of children with developmental disabilities had warmer, closer relationships with their brothers/sisters who had developmental disabilities compared to the brothers. Orsmond and Seltzer (2007) hypothesized that this gender difference may be due to the socialization process that encourages females to develop nurturing/caregiving behaviors more so than males. Additionally, the researchers noted that female siblings interacted with their siblings with disabilities more frequently and in a way that was more emotionally supportive. More frequent interaction and increased emotional support provided by the sibling is associated with a more effective learning environment for children with disabilities to develop knowledge of their feelings and the ability to regulate their own emotions (Stoneman, 2005).

Differing effects of sibling gender in relation to developing prosocial behavior may have specific implications in terms of providing additional resources for families with children diagnosed with ASD. Individuals with autism face extraordinary difficulties in reciprocating social-emotional experiences. Female siblings have a higher propensity for nurturing, supportive, and emotionally attentive interactions than their male counterparts; therefore, it is likely that their involvement in reciprocal emotional experiences will create opportunities for children with autism to develop necessary skills for regulating their own emotions (Kaminsky & Dewey, 2001). While male siblings can be a benefit to their siblings with autism, they may not engage in activities designed to provide direct support and regulation for the emotional well-being of their sibling (Rivers & Stoneman, 2008). Understanding how sibling gender impacts the relationship between prosocial behaviors and emotional regulation may provide researchers with meaningful data regarding the implementation of family-based interventions and help in designing support systems that take into account the optimal use of the positive aspects of the sibling relationship for children with autism.

### 1.1. Rationale of the Study

Examining the relationships among prosocial behaviors, parent styles, the dynamics of sibling gender, and the emotional regulation of children with Autism Spectrum Disorder (ASD) is an important area of research. Children with ASD are estimated to be approximately 1 in 54 in the U.S., and are increasing at alarming rates worldwide (Knopf, 2020). The way parents interact with their child with ASD can be greatly is related to their ability to regulate their emotions. Parents who use effective strategies such as keeping consistent routines, using supportive communication, and providing positive reinforcement are associated with a sense of stability and security, which is linked to the development of self-regulatory skills. Identifying the ways in which parents interact with their child with ASD and the associations between those interactions and their child’s ability to self-regulate will allow researchers to identify methods in which to intervene with children with ASD.

While there is considerable evidence that siblings of children with ASD contribute to the social and emotional development of children with ASD (Ben-Itzchak et al., 2019; Orm et al., 2022), little research has investigated how sibling characteristics (specifically, sibling gender) may influence the degree to which they contribute to their brother or sister’s emotional regulation. Gender socialization results in systematic differences in prosocial behavior and caregiving tendencies (Dittman et al., 2023); therefore, it is reasonable to suggest that the gender of the typically developing sibling could moderate the degree to which they contribute to their brother or sister’s emotional regulation. By examining a mediated moderation model, researchers can determine if authoritative parenting is associated with emotional regulation through promoting sibling prosocial behavior, and further, if this indirect path differs based on the gender of the sibling.

Through identifying successful parenting strategies and examining the role that siblings’ prosocial behaviors play, while also evaluating the possibility that sibling gender may serve as a moderator, clinicians and educators can establish comprehensive plans of action that utilize the strengths of family dynamics to promote the emotional regulation of children with ASD. This research can inform family-based interventions that optimize the contributions of siblings based on their natural strengths while building skills in areas requiring support. Ultimately, this may be associated with fewer behavioral problems, better social integration, and an improvement in the general well-being of children with ASD and their families.

### 1.2. Objectives of the Study

1. To examine the relationship between parenting practices and the emotional regulation of children with ASD.
2. To investigate the association between siblings’ prosocial behavior and the emotional regulation of children with ASD.
3. To explore the mediational role of siblings’ prosocial behavior on the association between parenting practices and emotional regulation of children with ASD.
4. To test a mediated moderation model examining whether sibling gender moderates the indirect effect of parenting practices on emotional regulation through prosocial behavior.

### 1.3. Hypotheses

**H1.**  *Parenting practices, such as authoritative parenting, have a positive relationship with emotion regulation, while authoritarian and permissive parenting styles have a negative relationship with emotional regulation in children with ASD.*

**H2.**  *The prosocial behavior of siblings has a positive relationship with the emotional regulation of children with ASD.*

**H3.**  *Prosocial behavior has a significant mediating effect on the relationship between parenting practices and emotional regulation.*

**H4.**  *Sibling gender moderates the indirect effect of authoritative parenting on emotional regulation through prosocial behavior (mediated moderation), with the indirect effect being stronger for female siblings.*

## 2. Method

### 2.1. Research Design

A correlation research design with mediated moderation analysis was used to examine the relationships between parental practices, the prosocial behavior of siblings, sibling gender, and emotional regulation of children with autism spectrum disorder.

### 2.2. Sample and Sampling

The study involved 600 parents/caregivers (*M* = 32.91, *SD* = 4.23) of children with ASD aged 25 to 40 years, selected through a non-probability purposive sampling technique from private rehabilitation centers in Lahore, Pakistan. Both male and female caregivers participated, representing various demographic characteristics such as age, gender, education, occupation, family system, monthly income and duration of therapy. Children with ASD aged 4 to 11 years with at least one older sibling aged 6 to 12 years, and children who were in therapy, were included. All children were enrolled through registered rehabilitation centers where formal ASD diagnoses had been established prior to participation. Diagnostic confirmation was therefore based on existing clinical records rather than reassessment within the study. Children with other disabilities or epilepsy and children outside the specified age range were excluded.

The study examined the demographic characteristics by means of a descriptive analysis. The children with ASD were almost evenly distributed by gender, with 305 (50.8%) females and 295 (49.2%) males. The age of the children with ASD varied, with the majority (322, 53.7%) falling in the age group 4–5, followed by 191 (31.8%) in the age group 6–7, 82 (13.7%) in the age group 8–9 and only 5 (0.8%) in the age group 10–11. The majority of mothers were housewives, while fathers had varying levels of education. The family system was predominantly joint (64%), with 36% belonging to nuclear family structures. The relationship with the child with ASD was predominantly between mothers and fathers, with 567 (94.5%) respondents being mothers. The order of birth of children with ASD was second, and the age of diagnosis varied. It is important to note that the majority of respondents were mothers (94.5%), reflecting the caregiving context in which mothers are typically the primary respondents for child-related assessments in this cultural setting.

The duration of therapy varied: 305 (50.8%) children received therapy for one year, 207 (34.5%) for two years, 66 (11.0%) for three years and 22 (3.7%) for four years. Among the siblings, 316 (52.7%) were female and 284 (47.3%) were male, with ages ranging from 6 to 12 years (*M* = 8.43, *SD* = 1.79). The distribution of sibling gender was relatively balanced across the sample.

### 2.3. Assessment Measures

The following instruments were used in this study.

Parenting Styles and Dimensions Scale (PSDQ), developed by Robinson et al. (2001), was used to assess parenting styles. It consists of 32 items and three subscales, including authoritative (15 items), authoritarian (12 items) and permissive parenting styles (5 items). Each item of the scale has a 5-point Likert scale, where 1 stands for never and 5 for always, and the Cronbach alpha reliability is α = 0.90. The Cronbach alpha reliability for the current study was α = 0.85. The PSDQ captures overall parenting practices within the family system and is not limited to maternal behaviors, making it suitable for use with mixed caregiver samples.

The Strengths and Difficulties Questionnaire (SDQ) by Goodman (1997) was used to assess emotional and behavioral problems. It comprises 4 subscales (peer relationship problems, conduct problems, prosocial behavior and hyperactivity/inattention) with 25 items and each item is rated on a three-point scale: 0 (not true), 1 (somewhat true) and 2 (certainly true). Its Cronbach’s alpha reliability is α = 0.87. In the current study, caregivers completed the SDQ with reference to the typically developing sibling (not the child with ASD), as required for examining siblings’ prosocial behavior as a mediator. Respondents were explicitly instructed: “Please answer the following questions about your older child who does not have autism.” Only the prosocial behavior subscale was used in the analysis. The prosocial subscale demonstrated acceptable internal consistency in this sample (Cronbach’s α = 0.76).

Emotional Regulation Checklist (ERC), developed by Shields and Cicchetti (1997) was used to measure the ability to regulate emotions. The Emotion Regulation Checklist included tests measuring socially acceptable emotional expressions, empathy, composure, and emotional understanding, among other adaptive regulation skills. Both construct validity and discriminant validity were demonstrated for the ERC. The Cronbach’s alpha of the current study was α = 0.76.

The Demographic Information Sheet was specially designed to collect information about participants’ age, gender, education, occupation, family system, monthly income, duration of therapy, and specifically, the gender and age of the sibling of the child with ASD.

### 2.4. Ethical Considerations

Ethical approval was initially granted by the Ethical Review Committee (ERC) at Riphah International University, Lahore. Prior to conducting the research, written consent was obtained from the authors of all measures. After official approval was granted by the head of the department, the purpose of the study was explained before participation. After the participants were willing to participate in the study, a consent form was signed, and they were properly informed of the objectives of the study and allowed to withdraw their participation at any time. In addition, participants were guaranteed the protection of the data they provided. The confidentiality and anonymity of participants were maintained throughout the study, and potential risks to participants were minimized.

### 2.5. Procedure

After approval of the research topic by the Board of Advanced Studies and Research, permission for the assessment measures was obtained from the original authors. Permission for data collection was obtained from the institutions involved. Prior to data collection, the researcher explained the nature and purpose of the study and invited them to participate in the research. After informed consent was obtained, the questionnaires were completed. Participants were given 15 and 20 min to complete the scales. All assessment measures were translated into Urdu using forward–backward translation procedures conducted by bilingual psychology researchers. The translated versions were reviewed to ensure conceptual equivalence and cultural appropriateness. Participants with sufficient English proficiency were offered the English versions of the measures. The researcher was available to clarify items in both languages, following a standardized protocol to maintain consistency across participants. After completing the questionnaires, the researcher went through the questionnaires to see if any questions remained unanswered. If so, the researcher asked the participant to complete the information in the appropriate section of the questionnaire. Finally, the researcher thanked the participants for their voluntary participation in the study.

## 3. Results

The data were analyzed with the help of IBM SPSS 23. The proposed hypotheses were evaluated using Pearson Product-Moment Correlation Analysis to assess the relationship between the variables, Simple Mediation Analysis using PROCESS Macro Model 4 to examine the mediation effect of the prosocial behavior of siblings between parenting practices and emotional regulation in children with ASD, and Moderated Mediation Analysis using PROCESS Macro Model 14 to test whether sibling gender moderates the indirect effect of parenting practices on emotional regulation through prosocial behavior.

### 3.1. Correlation Analysis

Pearson product-moment correlation analysis was used with SPSS to determine the relationship between parenting practices, emotional regulation, and prosocial behavior of siblings in children with ASD.

Table 1 reveals that authoritative parenting practices demonstrated a significant positive relationship with emotional regulation in children with ASD (*r* = 0.46, *p* < 0.01). The prosocial behavior of siblings was also positively correlated with emotional regulation (*r* = 0.23, *p* < 0.05). However, neither authoritarian parenting (*r* = 0.02, *p* > 0.05) nor permissive parenting (*r* = 0.04, *p* > 0.05) showed significant associations with emotional regulation in this sample. The prosocial behavior of siblings was also positively correlated with emotional regulation.

### 3.2. Mediation Analysis

Mediation analysis used PROCESS Model 4 (Hayes, 2018) with 5000 bootstrap samples and bias-corrected 95% confidence intervals. No covariates were included. The model tested whether siblings’ prosocial behavior (M) mediates the relationship between authoritative parenting (X) and emotional regulation (Y) through three regression equations: X→M (path *a*), X + M→Y (paths *c’* and *b*), and X→Y (path *c*). All coefficients are unstandardized (*B*).

Table 2 revealed that the direct effect of authoritative parenting style on emotion regulation in children with ASD was statistically significant and also significantly related to the prosocial behavior of siblings on emotion regulation. The indirect effect of authoritative parenting style on emotional regulation through the prosocial behavior of siblings was statistically significant. The mediation model accounted for 23% of variance in emotional regulation (R^2^ = 0.23). Bootstrap confidence intervals (5000 samples) confirmed the significance of the indirect effect. This suggests that the prosocial behavior of siblings plays a partially mediating role between authoritative parenting and emotional regulation in children with ASD.

**Figure 1:**
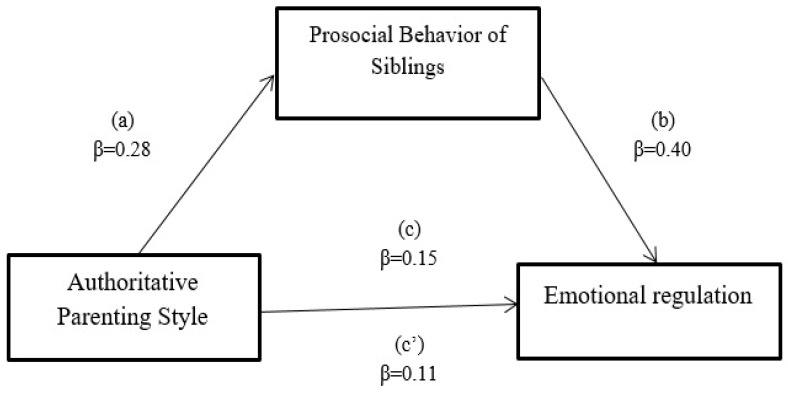
Path Analysis Model of Associations Between Authoritative Parenting, Emotional Regulation and Prosocial Behavior of Siblings.

Figure 1 indicates that the prosocial behavior of siblings partially mediates the relationship between authoritative parenting and emotional regulation in children with ASD.

### 3.3. Mediated Moderation Analysis

**Figure 2:**
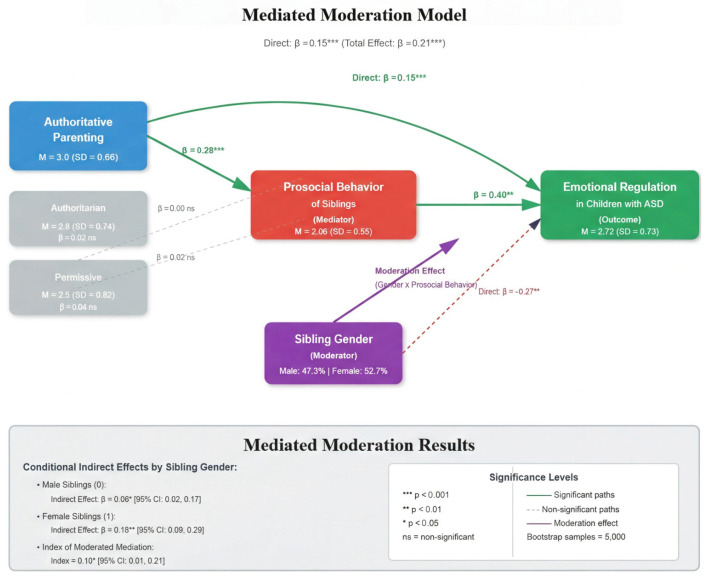
Mediated Moderation Model of Authoritative Parenting (exogenous variable) and Prosocial Behavior, Emotional Regulation, and Sibling Gender (endogenous variables).

The mediated moderation analysis was conducted using Hayes PROCESS Macro Model 14 with 5000 bootstrapped samples to examine whether sibling gender moderates the indirect effect of authoritative parenting on emotional regulation through prosocial behavior. Sibling gender was coded as 0 = male and 1 = female. The conceptual model is depicted in Figure 2, and the results are presented in Table 3.

Critically, the interaction between prosocial behavior and sibling gender significantly predicted emotional regulation (*B* = 0.12, *SE* = 0.05, *t* = 2.40, *p* = 0.02), indicating that sibling gender moderates the relationship between prosocial behavior and emotional regulation. The overall model accounted for 23% of variance in emotional regulation (R^2^ = 0.23). The index of moderated mediation was significant (Index = 0.10, 95% CI [0.01, 0.21]), confirming that sibling gender moderates the strength of the indirect pathway from authoritative parenting to emotional regulation through prosocial behavior.

Conditional indirect effects revealed that this pathway was significantly stronger for female siblings (*B* = 0.18, 95% CI [0.09, 0.29]) compared to male siblings (*B* = 0.08, 95% CI [0.02, 0.17]). Both conditional indirect effects were statistically significant, as confidence intervals did not include zero. This indicates that while prosocial behavior mediates the relationship between authoritative parenting and emotional regulation for siblings of both genders, this mediating effect is approximately twice as strong when the sibling is female.

Figure 2 illustrates the mediated moderation model showing that authoritative parenting influences emotional regulation in children with ASD both directly and indirectly through siblings’ prosocial behavior. The indirect pathway is significantly moderated by sibling gender, with female siblings demonstrating stronger facilitation of emotional regulation through prosocial behaviors compared to male siblings.

Although the primary models did not include demographic covariates, post hoc sensitivity analyses controlling for child age and therapy duration yielded a comparable pattern of results, suggesting that the observed indirect and moderated effects were not solely attributable to these variables.

## 4. Discussion

The results from the current study are presented below in the context of previous studies that examined the relationships among parenting practices, sibling prosocial behavior, sibling gender, and emotional regulation in children with Autism Spectrum Disorder (ASD).

As predicted, Hypothesis 1 received partial support. The relationship between authoritative parenting practices and children with ASD’s emotional regulation was found to be positively related, which is consistent with previous research. Research has shown that authoritative Iranian mothers were found to have greater use of emotion regulation strategies, including suppression and reappraisal, as compared to mothers who used either permissive or authoritarian styles (Bahrami et al., 2018). It appears as though authoritative parenting provides a structured environment for developing emotional regulation through role-modelling, structuring and emotional coaching (Karst & Van Hecke, 2012).

Contrary to what was expected, however, neither authoritarian nor permissive parenting styles were found to have a significant relationship with children with ASD’s emotional regulation in this study. There are several possible explanations for why this was not the case: the specialized needs of children with ASD may limit or mitigate the association seen in studies examining neurotypical populations (Cibralic et al., 2019) or the relatively low endorsement of these parenting styles in our sample may have limited statistical power to detect relationships. Additionally, cultural factors in the Pakistani context, including beliefs about child development and varying levels of autism awareness among families (Minhas et al., 2015), may influence how different parenting practices manifest and affect child outcomes. Future research should examine these parenting styles with larger, more diverse samples and consider cultural moderators.

The second hypothesis, which hypothesized a positive relationship between sibling prosocial behavior and emotional regulation in children with ASD, was also supported. However, the relationship between sibling prosocial behavior and emotional regulation in children with ASD was impacted by the distinct challenges in the family dynamics of children with ASD. Siblings of children with ASD may have fewer prosocial interactions with their siblings compared to siblings of children with physical disabilities due to differences in prosocial behavior exhibited by children with ASD and difficulty reciprocating socially (Orm et al., 2022). Children with ASD also require an understanding of specific subtleties related to having a child with ASD, i.e., predictable needs and an insistence on sameness, as opposed to the care requirements of a child with a physical disability, such as feeding or mobility assistance. Finally, children with ASD have been shown to have a higher incidence of the broader autism phenotype and consequently exhibit higher levels of autism traits, which have been associated with poorer prosocial behavioral profiles (Chao, 2000). Regardless of the challenges, however, the results from this study indicate that sibling prosocial behavior continues to remain a significant contributor to emotional regulation.

The third hypothesis that proposed sibling prosocial behavior would act as a mediator in the relation between parents’ practice and children’s emotional regulation was supported. Research findings show that authoritative parenting has both direct associations on children’s emotional regulation and indirect associations through creating an environment where children will develop prosocial behaviors toward their siblings, and according to family systems theory (Bronfenbrenner, 1979), the family system as a whole is the source of a child’s social environment, and it is the interactions among all the members of the family system that creates a social environment for the child. The mediation model shows that authoritative parenting is associated with a family environment that encourages prosocial behavior in typically developing siblings; this can provide an additional resource for a child with autism to learn how to regulate their emotions. The indirect way in which the parenting practices are associated with children’s emotional regulation suggests that the positive effects of authoritative parenting occur through a number of mechanisms, including the direct parent–child relationship, but also indirectly through the child’s relationships with his/her siblings. Although the partial mediation indicates that sibling prosocial behavior is an important mediator of the relation between authoritative parenting and children’s emotional regulation, authoritative parenting also directly contributes to children’s emotional regulation through modeling, providing children with structure and emotional coaching. Thus, authoritative parenting may be a multifaceted way in which parents contribute to the development of their children’s emotional regulation abilities. Similar patterns of psychological influence from sociocultural environments on emotional and identity development have been documented in recent research examining self-perception and body image in emerging adults (Arshad et al., 2025).

The fourth hypothesis of mediated moderation was supported. The index of mediated moderation was significant, showing that the type of sibling (male or female) modulated the strength of the indirect pathway from authoritative parenting to children’s emotional regulation through sibling prosocial behavior. The indirect effect of authoritative parenting on children’s emotional regulation via prosocial behavior was greater for sisters than for brothers, although both indirect effects were statistically significant. These results provide further evidence that the mechanisms through which parenting practices are translated into children’s emotional regulation are more complex than previously thought, and are influenced by the sex composition of the sibling dyad.

The present research builds upon the work of Dittman et al. (2023), who found that gender socialization processes influence how children of different sexes interact with family members with special needs. The current study demonstrates empirically that the gender-based interaction patterns in sibling relationships, found by Dittman et al. (2023), are associated with children’s emotional regulation outcomes in children with autism spectrum disorder. The findings support Kaminsky and Dewey’s (2001) conclusion that sibling relationships in families with children with autism are shaped by many factors, including the sex of the siblings, which can either enhance or impede the quality and effectiveness of the supportive role that each sibling plays in supporting the child with autism. The finding of mediated moderation provides additional support for family systems theory, which emphasizes the interrelatedness of family relationships and the necessity of examining the characteristics of multiple family members when attempting to understand developmental outcomes. By showing that the relationship between parents’ practices and child outcomes occurs through the sibling’s prosocial behavior and is moderated by the gender of the sibling, this research highlights the complexity of family influences on children with autism.

### 4.1. Limitations

While there are some limitations to the present study, it is possible to consider a few in greater detail. Because this study used a purposive sampling strategy, the results cannot be assumed to apply to every family type. In addition, due to its cross-sectional design, the study was unable to determine the direction of causation; therefore, it is unclear if increased use of sibling-prosocial behavior (or reduced use of sibling-negative behavior) is responsible for improvements in the emotional regulation of children with ASD, or if it is simply the result of them having improved emotional regulation. The lack of statistical control for socioeconomic indicators, family structure, educational context, peer relationships, and caregiving burden constrains causal inference and suggests that the observed gender-related differences may, in part, be attributable to unmeasured sociocultural or structural influences rather than sibling gender itself.

It is also important to note that the study did not control for extrinsic variables that may affect a child’s ability to emotionally regulate, including their educational setting, peer relationships, etc., and the various professional therapies available to support the child and family. Additionally, the exclusive reliance on parent-report questionnaires introduces common-method variance that may inflate observed associations. Another limitation is that the majority of respondents were mothers, which may limit the generalizability of findings to paternal parenting practices. Although the construct of parenting practices reflects household-level caregiving, future research should include more balanced maternal and paternal samples to examine potential parent-gender differences.

Another area in which this study is limited is that it did not assess the gender of the child with ASD relative to the gender of their sibling, which could influence the dynamics that were observed. Therefore, one area that future research should investigate is whether the gender of the siblings (same-sex vs. opposite-sex) affects the degree to which the siblings’ prosocial behaviors impact the child with ASD’s ability to emotionally regulate.

In addition, the study did not evaluate other factors that could affect the relationships among these variables, including the age difference between the siblings, birth order, and the overall quality of the relationship between the siblings, which may interact with the gender of the siblings to produce different outcomes.

### 4.2. Future Recommendations of the Study

To address single-method bias, future studies should incorporate multi-informant reports (e.g., teachers, clinicians) and behavioral observations alongside parent questionnaires. Future studies must involve participants from more diverse backgrounds to make findings more generalizable, use behaviorally measured assessments to limit the bias associated with self-reported data, and conduct longitudinal studies to assess changes in parent’s behavior and child’s emotional regulation across time. In addition to assessing how programs to increase prosocial behaviors in siblings are effective, the research must also assess how parents encourage prosocial behaviors in their siblings.

To fully understand how emotional regulation is developed in children with ASD, further research will need to address the influence of peer relationships, school based resources and professional therapeutic interventions. Research must assess gender specific effects of sibling relationships on the impact of prosocial behaviors on reducing symptoms of emotional dysregulation. To achieve this, researchers must test interactions between moderating variables such as sibling gender, age difference and relationship quality, using more advanced statistical techniques. Longitudinal studies examining sibling relationships during early childhood and adolescence may also determine how differences in emotional regulation due to gender evolve over time.

In addition to quantitative studies, qualitative research assessing the subjective experience of male and female siblings of children with ASD may also provide additional context to support the findings of quantitative studies. Finally, research must focus on developing and testing support programs for siblings of children with ASD that take into account the gender of the sibling and provide each gender of the sibling opportunities to use their unique strengths. Programs like this would allow male siblings to develop emotionally supportive skills, and assist female siblings in developing skills to manage their potential caregiving responsibilities which can aid in emotional regulation of children with ASD.

### 4.3. Future Implications of the Study

This research also has many implications for clinicians, teachers, education professionals and parents who have children with ASD by providing an understanding of parent–child and sibling interactions that will help develop interventions that are effective for children with ASD. Clinicians may use family-based therapy techniques, school professionals can create and implement structured programs, and parents can receive education/training in the practice of authoritative parenting. Community agencies may also create parent-support groups to provide opportunities for shared learning and emotional support among families of children with ASD.

Additionally, the study’s mediated moderation findings emphasize the importance of taking into consideration sibling gender in family-based interventions since the gender of the sibling affects how prosocial behavior contributes to emotional regulation in children with ASD. However, our findings suggest potential value in considering sibling characteristics when designing family interventions, though this should be approached cautiously. Rather than prescriptive gender-based programming, practitioners might assess individual sibling strengths and needs while remaining aware that gender-related patterns may emerge on average. Interventions should not reinforce stereotypes while acknowledging that siblings may require different forms of support based on their individual strengths, developmental stages, relationship qualities, and broader family environments. Without direct measures of caregiving roles, interaction patterns, or the mechanisms behind gender differences, recommendations for interventions should focus on assessing individuals rather than categorically using gender-based approaches. Parent-training programs should provide parents with education regarding the diversity of sibling interactions to enhance prosocial behavior in all children, and avoid prescribing rigid or stereotypical role expectations for each child.

## 5. Conclusions

The relationships among child-rearing practices, prosocial behaviors among siblings, gender of the siblings, and how children with ASD regulate their emotions is very complex, which will make it a good basis for developing family-based interventions. In this study, it was shown that an authoritative approach to parenting promotes emotional regulation, which was also supported by the prosocial behaviors exhibited by the sibling(s), and the indirect influence of prosocial behaviors is greater for female siblings than for male siblings. Therefore, the present findings demonstrate that when designing interventions for children with ASD, the whole family unit needs to be considered, and further studies are needed to better understand the dynamics of families so that family-based interventions can be developed to effectively capitalize on family strengths in promoting emotional regulation of children with ASD.

## Figures and Tables

**Table 1:** Descriptive Statistics and Correlations for Study Variables.

	Variables	*M*	*SD*	2	3	4	5
	Parenting Practices						
1	Authoritative	3.0	0.86	0.06	0.17	0.40 **	0.46 **
2	Authoritarian	2.8	0.74	-	0.48 **	0.09	0.02
3	Permissive	2.5	0.62	-	-	0.02	0.04
4	Prosocial behavior	2.06	0.55	-	--	-	0.23 *
5	Emotional regulation	2.72	0.73	-	-	-	-

*Note.* * *p* < 0.05. ** *p* < 0.01.

**Table 2:** Mediation Effect of Siblings’ Prosocial Behavior on Association Between Parenting Practices and Emotional Regulation Among Children with ASD.

Paths	*B*	*SE*	*T*	*p*	95% CI	
					*LL*	*UL*
Authoritative → Parenting Prosocial behavior	0.28	0.05	5.60	0.001	0.18	0.38
Prosocial behavior → Emotional regulation	0.40	0.12	3.33	0.002	0.16	0.64
Authoritative Parenting → Emotion regulation	0.15	0.04	3.75	0.001	0.07	0.23
Effect						
Direct	0.15	0.04	3.75	0.001	0.07	0.23
Indirect	0.11	0.03		0.004	0.05	0.18
Total	0.26	0.04	6.50	0.001	0.18	0.34

**Table 3:** Path Coefficients for Mediated Moderation of Sibling Gender.

Probing Moderated Indirect Relationships			95% CI	
	Unstandardized Coefficient	*SE*	*LL*	*UL*
Prosocial Behavior × Gender → Emotion Regulation	0.12	0.05	0.02	0.22
Sibling Gender: Male (0)	0.08	0.04	0.02	0.17
Sibling Gender: Female (1)	0.18	0.05	0.09	0.29
Index of Mediated Moderation	0.10	0.05	0.01	0.21

*Note.* CI = Confidence Interval, *LL* = Lower Limit, *UL* = Upper Limit. Bootstrap samples = 5000. Confidence intervals that do not include zero indicate significant effects.

## Data Availability

The data collected for this study have been used within this article. However, the complete set of data is available on request from the corresponding author.
